# Genomic profiling supports the diagnosis of primary ciliary dyskinesia and reveals novel candidate genes and genetic variants

**DOI:** 10.1371/journal.pone.0205422

**Published:** 2018-10-09

**Authors:** Marina Andjelkovic, Predrag Minic, Misa Vreca, Maja Stojiljkovic, Anita Skakic, Aleksandar Sovtic, Milan Rodic, Vesna Skodric-Trifunovic, Nina Maric, Jelena Visekruna, Vesna Spasovski, Sonja Pavlovic

**Affiliations:** 1 Laboratory for Molecular Biomedicine, Institute of Molecular Genetics and Genetic Engineering, University of Belgrade, Belgrade, Serbia; 2 Mother and Child Health Care Institute of Serbia „Dr Vukan Cupic“, Belgrade, Serbia; 3 School of Medicine, University of Belgrade, Belgrade, Serbia; 4 Clinic for Pulmonology, Clinical Center of Serbia, Belgradе, Serbia; 5 Clinic for children diseases, University Clinical Center of the Republic of Srpska, Banja Luka, Bosnia and Herzegovina; University of Toronto, CANADA

## Abstract

Primary ciliary dyskinesia (PCD) is a rare inherited autosomal recessive or X-linked disorder that mainly affects lungs. Dysfunction of respiratory cilia causes symptoms such as chronic rhinosinusitis, coughing, rhinitis, conductive hearing loss and recurrent lung infections with bronchiectasis. It is now well known that pathogenic genetic changes lead to ciliary dysfunction. Here we report usage of clinical-exome based NGS approach in order to reveal underlying genetic causes in cohort of 21 patient with diagnosis of PCD. By detecting 18 (12 novel) potentially pathogenic genetic variants, we established the genetic cause of 11 (9 unrelated) patients. Genetic variants were detected in six PCD disease-causing genes, as well as in *SPAG16* and *SPAG17* genes, that were not detected in PCD patients so far, but were related to some symptoms of PCD. The most frequently mutated gene in our cohort was *DNAH5* (27.77%). Identified variants were in homozygous, compound heterozygous and trans-heterozygous state. For detailed characterization of one novel homozygous genetic variant in *DNAI1* gene (c. 947_948insG, p. Thr318TyrfsTer11), RT-qPCR and Western Blot analysis were performed. Molecular diagnostic approach applied in this study enables analysis of 29 PCD disease-causing and related genes. It resulted in mutation detection rate of 50% and enabled discovery of twelve novel mutations and pointed two possible novel PCD candidate genes.

## Introduction

Primary ciliary dyskinesia (PCD (OMIM #244400)) is a rare inherited autosomal recessive or X-linked disorder, which affects lungs, reproductive organs, and organ laterality. Major characteristics of PCD are ultrastructural defects of cilia leading to ciliary immotility or abnormal ciliary motility [1, 2]. PCD presents with neonatal respiratory distress in 80% of cases, recurring acute rhinosinusitis [3], rhinitis [4], sinusitis, conductive hearing loss due to otitis media, recurrent or chronic lung infections with bronchiectasis and progressively declining lung function[5]. Since motile cilia are present throughout the respiratory tract, every loss of structural/functional integrity of cilia leads to disorder in the primary innate defense mechanism of mucociliary clearance [6]. Around 50% of PCD patients have *situs inversus* (SI) [3], and reduced fertility in males and females is observed [7, 8]. The estimated prevalence of PCD is 1 in 15.000–30.000 live births, but recent studies propose that prevalence is higher in consanguineous populations[9]. Early recognition of PCD and the correct diagnosis are often delayed due to the clinical symptoms overlapping with other chronic airway disorders.

The clinical features of PCD are nowadays well recognized, but the diagnosis is still challenging, especially when patients represent non-specific signs and symptoms, and when necessary equipment and screening tests including nasal nitric oxide (nNO) measurements, nasal mucociliary transport tests, and saccharine test, are not available to physicians. Even transmission electron microscopy, which is considered as “gold standard” for PCD, cannot resolve 30% of PCD patients with normal cilia ultrastructure [10–15]. Therefore, implementation of genetic and molecular diagnosis of PCD is rather a necessity, regardless of whether it is a confirmation of a clinical diagnosis or suspicion of PCD. The identification of PCD genes has been based on linkage studies, candidate gene approaches and proteomic analyses, combined with sequencing of potentially causative genes [16–19]. Recently, the availability of high-throughput sequencing techniques contributed to the swift identification of new PCD-causative genes, resulting in more than 40 genes to be identified so far, which allowed 65% of cases to be genetically described [15, 20–23].

Herein, we analyzed the cohort of clinically suspected PCD patients from Serbia using a Clinical-Exome Next-Generation Sequencing (NGS). Using this approach we confirmed some genetic variants already classified as disease-causing mutations, and discovered 12 novel genetic variants that had never been reported to be associated with PCD. In this study we also describe functional characterization of the novel, potentially pathogenic variant in the *DNAI1 (CILD1)* gene found in two siblings.

## Materials and methods

### Subjects

This study has been approved by the Ethics Committee of the Mother and Child Health Care Institute of Serbia Dr Vukan Cupic, Belgrade, Serbia. The study has been performed in accordance with the ethical standards lain down in the Helsinki Declaration and its later amendments. Informed consent was obtained from all participants included in the study.

Inclusion criteria for PCD diagnosis included symptoms such as neonatal respiratory distress, chronic sinusitis, bronchiectasis, recurrent pneumonia and SI. For identification of cilia motility, respiratory epithelial cells were obtained by nasal brushing, and cilia movements were detected using optical microscope. Exclusion criteria implied the absence of cystic fibrosis (CF), for which purpose patients were tested for presence of *Pseudomonas aeruginosa* and sweat chloride test.

Extensive genetic analysis on 21 subjects from 18 unrelated families was conducted. Control group was consisted of in-house TruSight One collection of 69 subjects from general population of Serbia. Co-segregation analysis was performed for three families. For that purpose parents from these families as well as a child suspected to have PCD from third family, were recruited and tested by Sanger sequencing.

Genomic DNA was isolated from peripheral blood by a salting-out method. The samples were then referred to the Institute of Molecular Genetics and Genetic Engineering (IMGGE) for genetic profiling. The quality and quantity of isolated DNA was measured using Qubit 3.0 fluorimeter (Invitrogen, USA).

### Genetic and Bioinformatic analysis

Twenty one patients were analyzed by NGS approach using the Illumina Clinical-Exome Sequencing TruSight One Gene Panel. This panel includes all the known disease-associated genes described in the OMIM database until 2013, designed to cover all exons and flanking intronic regions of 4813 genes (~ 62.000 exons). 5ng/ul (10ul) of genomic DNA was used in all reactions, and quantification of DNA in all steps of NGS sequencing was determined using Qubit 3.0 fluorimeter (Invitrogen, USA). Qubit dsDNA HS Assay Kit with range of 0.2-100ng was used (Invitrogen, USA). Illumina MiSeq platform was used to generate libraries and to produce the VCF files. The software for analyzing data gained using NGS approach was IlluminaVariant Studio 2.0 and 3.0 (Illumina, San Diego, USA). VCF file contained approximately 8–10.000 variants per sample. After annotation of all samples we have accessed to variant filtering. The Filters section within IlluminaVariant Studio contains multiple parameters from which we have used the following: 1. General section: genotype-all, variant type-all, chromosomes-all; 2. Variant section: Pass Filter, 3. Consequence section: ClinVar pathogenic, Show only variants that are-all (missense, frameshift, stop gained, stop lost, initiator codon, in-frame insertion, in-frame deletion, splice); 4. Population frequency section: Thousand Genomes: set all frequencies on less than 5%, Exac: set all frequencies on less than 5%; 5. Classification section: Filter by classification-presumed pathogenic, pathogenic, unknown significance. After applying these filters, we got around 100 variants per sample. Than we prioritized 29 genes of interest for PCD (disease-causing genes according to OMIM, candidate genes coding for proteins which directly interacts with PCD disease-associated proteins according to String Interaction Network (https://string-db.org/), and genes belonging to the same gene family as disease-causing genes) (Table 1), and got three to five variants per sample. Variants that had allele frequencies less than 5% (according to 1000 Genomes and ExaC), predicted as damaging (according to SIFT and PolyPhen2 which are implemented into VariantStudio), and absent in our in-house TruSight One collection of 69 samples sequenced with the same methodology, were further analyzed and considered as candidate variants (one or two per sample). In-house TruSight One base served for exclusion of population specific variants. If some variant had higher frequency than 5% in European population and predicted as pathogenic, but wasn’t detected in our patients’ cohort, we took it into consideration.

**Table 1:** A list of analyzed PCD-related genes.

PCD causative genes	Protein localization/function	Variants detected in our study	The incidence of variants in our study
***CCDC39***	N-DRC	-	0%
***CCDC40***	N-DRC	+	11.11%
***CCDC103***	Cytoplasmatic, ODA assembling	-	0%
***DNAAF1 (LRRC50)***	Cytoplasmatic, DA assembling	-	0%
***DNAAF2 (KTU)***	Cytoplasmatic, DA assembling	-	0%
***DNAAF3***	Cytoplasmatic, DA assembling	-	0%
***DNAI1***	ODA	+	16.66%
***DNAI2***	ODA	-	0%
***DNAL1***	ODA	+	16.66%
***DNAH1***	ODA	-	0%
***DNAH5***	ODA	+	27.77%
***DNAH9***	ODA	-	0%
***DNAH11***	ODA	+	5.55%
***HEATR2***	Cytoplasmatic, DA assembling	-	0%
***HYDIN***	CP	-	0%
***LRRC6***	Cytoplasmatic, DA assembling	+	5.55%
***NME8 (TXNDC3)***	ODA	+	0%
***OFD1***	Cytoplasmatic	-	0%
***RPGR***	Cytoplasmatic	-	0%
***RSPH4A***	RS	-	0%
***RSPH9***	RS	-	0%
**PCD candidate genes**			
***CCDC8***	Regulate microtubule dynamics	-	0%
***CCDC50***	Associates with microtubule-based structures	-	0%
***CCDC88***	Role in ciliogenesis and cilium morphology	-	0%
***DYNC1H1***	Microtubule motor activity	-	0%
***NME1***	Centrosome	-	0%
***SPAG16***	Role in motile ciliogenesis	+	11.11%
***SPAG17***	Proper function of the axoneme	+	5.55%
***TCTE1***	Component of axonemal dynein and cytoplasmic dynein 1	-	0%

CP, central pair; DA, dynein arms; ENaC, amiloride-sensitive epithelial sodium channel; N-DRC, nexin-dynein regulatory complex; ODA, outer dynein arm; RS, radial spoke.

### Mutation validation and co-segregation analysis

The following databases were used to additionally determine candidate variants: VarSome (which includes the following databases: gnomAD genomes, gnomAD exomes, ClinVar, 1000 Genomes Project, and classification according to American College of Medical Genetics and Genomics (ACMG)), dbSNP, Exome Aggregation Consortium (ExaC), Ensembl, and The Human Gene Mutation Database (HGMD). Prediction tools used for pathogenicity scoring were as follows: Deleterious Annotation of Genetic Variants Using Neural Networks (DANN), MutationTaster, and Functional Analysis through Hidden Markov Models (FATHMM-MKL). Effects on protein level were investigated with SIFT, Provean, and PolyPhen2 in silico tools.

We used I-TASSER server (http://zhanglab.ccmb.med.umich.edu/I-TASSER/) to generate a PDB files and for identification of amino acids required for protein-protein interactions (PPIs). For analysis of protein structure, we used UCFS Chimera tool (http://www.rbvi.ucsf.edu/chimera).

The results obtained by NGS method were further verified by direct sequencing using the Big Dye terminator cycle sequencing kit and the ABI PRISM 310 automated sequencer (Applied Biosystems, USA). Primer sequences are listed in S1 Table. Co-segregation analysis was performed for three families which were available for the analysis.

### RNA extraction and synthesis of cDNA

For extraction of total RNA peripheral blood mononuclear cells from two probands, their parents, and 11 healthy controls were purified on Ficoll-PaqueTM Plus (GE Healthcare, USA) density gradient. TRI Reagent Solution (Ambion, USA) was used for isolation of total RNA, according to the manufacturer’s protocol. Complementary DNA (cDNA) was synthesized from 1μg of total RNA using RevertAid M-MuLV Reverse Transcriptase (ThermoFisher Scientific, USA).

### Relative quantification (RT-qPCR)

Expression of mRNA was determined using KAPA PROBE and SYBR Green Universal qPCR kit (KAPA Biosystems, USA). Real-time PCR was performed using ABI 7500 Real-Time PCR System (Applied Biosystems, USA). The Glyceraldehyde 3-phosphate dehydrogenase gene (*GAPDH*) was used as an endogenous control and median expression level of the healthy control group was used as calibrator. Relative quantification analysis was performed by a comparative ddCT method. All experiments were performed in duplicates. Primers for DNAI1 transcript designed according to instructions listed in [24], and their sequences are listed in S2 Table.

### Western blot analysis

Peripheral blood from two patients with the novel genetic variant, their parents, and healthy control was used for isolation of total cellular proteins for Western blot analysis. Peripheral blood cells were lysed with Lysis Buffer, treated with TEN (Tris-EDTA-NaCl) containing protease inhibitor (Roche, Switzerland) in 0.25M TrisHCl using three freeze- and- thaw cycles. For antibody validation, proteins from Hek293 cell line were used as negative control. DNAI1 protein was detected by Western blot using rabbit Anti-Dynein intermediate chain 1 monoclonal antibody (Abcam, United Kingdom), anti-rabbit IgG horseradish peroxidase conjugate (GE Healthcare, USA) and the enhanced chemiluminescence detection system (GE Healthcare, USA).

## Results

### Clinical phenotype and mutation profiling

Study included 21 subjects, 10 males (47.60%) and 11 females (52.40%) with an age range of 1 to 35 years, and two family members (parents). *Situs solitus* was observed in 11/21 (52.4%) patients, *situs inversus* was present in 10/21 (47.60%) patients, whereas *situs ambiguous* was not detected. Neonatal respiratory distress was present in 17/21 (80.95%), 19/21 had chronic sinusitis (90.47%), 17/21 had bronchiectasis (80.95%), and 9/21 had recurrent pneumonia (42.86%). Microscopically observed immotile cilia was present in 17/21 (80.95%), 4/21 patients had motile cilia (19.05%). In the group of patients with motile cilia 1/4 (25.00%) had normal ciliary beat pattern, whereas 3/4 had pathological ciliary beat pattern (Table 2). All patients were negative for presence of *Pseudomonas aeruginosa*, and concentration of chloride in sweat was not elevated. Additionally, detected symptoms were chronic secretory otitis media and haemoptysis. Parents of all analyzed patients had confirmed that consanguinity is not present in their families.

**Table 2:** Clinical and genetic characteristics of analysed patients in population of Serbia.

	Patients
	21 (100.00%)
Males	11 (52.40%)
Females	10 (47.60%)
**Diagnostic procedures**	
**Symptoms**	
Bronchiectasis	17 (80.95%)
Chronic sinusitis	19 (90.47%)
Neonatal respiratory distress	17 (80.95%)
Recurrent pneumonia	9 (42.86%)
**Organ laterality**	
*Situs inversus*	10 (47.60%)
*Situs solitus*	11 (52.40%)
*Situs ambiguous*	0
**Cilia motility**	
**Motile:**	4 (19.05%)
Normally ciliary beat pattern	1/4 (25.00%)
Pathological ciliary beat pattern	3/4 (75.00%)
Non-motile	17 (80.95%)
**Genetics**	
Mutations detected in PCD causative and candidate genes	9 (50%)
Homozygous mutations in *CFTR* gene	0

In order to determine the genetic basis of 21 patients suspected to have PCD, we used TruSight One Gene Panel and generated comprehensive libraries. We prioritized 29 genes listed in Table 1, and used the recessive disease model which included homozygotes, compound heterozygotes and trans-heterozygotes. Analysis of the 29 genes revealed a total of 2210 variants. Then we searched for this variants within in-house TruSight One base and discarded the variants that also exist in general population of Serbia for further analysis. Overall, we have detected 18 different pathogenic genetic variants in PCD disease-causing and candidate genes in 11 patients (9 unrelated patients), which may be considered as potential genetic causes of the disease, since various prediction tools suggested their pathogenicity. Among 9 PCD patients, homozygous variants were detected in 3 patients, 4 carried compound heterozygous variants, and 2 had trans-heterozygous variants. In 3 of 4 patients with compound heterozygous variants, the inheritance was not confirmed due to the unavailability of the parental samples. Disease-causing variants were detected in *CCDC40*, *DNAI1*, *DNAL1*, *DNAH5*, *DNAH11*, *LRRC6*, *SPAG16* and *SPAG17* genes (Table 3). The most frequently mutated alleles were within *DNAH5* gene (27.77%) and none of 21 analysed patient had homozygous mutation in *CFTR* gene. We also included three monoallelic variants in Table 3, (variant c.8999G>A in *DNAH5* gene; variant c.27T>G, in *LRCC6* gene; and variant c.8555C>G, in *DNAH11* gene,) which were detected in our patients, and shown to be potentially pathogenic, but these patients were not included in the above-mentioned 9 PCD patients.

**Table 3:** Spectrum of detected genetic variants in Serbian patients with PCD.

GENE	dbSNP ID	Genomic coordinates	Genetic variants		Allele freq Eur (%)^a^	ACMG classification^b^	DANN^c^	SIFT/Provean^d^	Patient ID	Reference from HGMD/ClinVar
			Nucleotide change	Amino acid change						
***CCDC40***	rs397515393	chr17-78013765	c. 248delC	p.Ala83ValfsTer84	0	Pathogenic (PVS1, PS3)	NA	Not applicable	P5	[25]
	rs747233125	chr17-78060006	c. 2440C>T	p.Arg814Ter	0	Pathogenic (PVS1, PM2, PM4, PP2)	0.9899	Damaging	P13, P14	[26]
***DNAI1***	-	chr9-34500765	c. 947_948insG	p.Thr318TyrfsTer11	unknown	NA^e^	NA	Not applicable	P9, P10	novel
	rs867262419	chr9-34512140	c.1345_1349delCTTAA	p.Asn450LeufsTer6	0	NA^e^	NA	Not applicable	P21	novel
	-	chr9-34514506	c.1684G>A	p.Asp562Asn	0	Uncertain Significance (PM2, PP3)	0.9993	Damaging	P21	novel
***DNAH5***	-	chr5-13864748	c. 4356-2A>G	-	0	Pathogenic (PVS1, PM2, PP3)	0.9949	Not applicable	P4	novel
	-	chr5-13809281	c.7624T>C	p.Trp2542Arg	0	Uncertain Significance (PM2, PP3)	0.9919	Damaging	P4	novel
	rs137949961	chr5-13777417	c.8999G>A	p.Arg3000Gln	0.26	Uncertain Significance (PP3)	0.9989	Damaging	P6	ClinVar 219734
	rs140782270	chr5-13914743	c.1206T>A	p.Asn402Lys	0.13	Uncertain Significance (PM2, PP3)	0.9979	Damaging	P20	ClinVar 188080
	-	chr 5–13718980	c.8012A>G	p.Gln2701Arg	0	NA^e^	NA	Damaging	P20	novel
***DNAL1***	rs751754576	chr14-74154044	c.347A>T	p.Lys116IIe	0	Uncertain Significance (PM2, PP3)	0.9926	Damaging	P1	novel
	rs535885451	chr14-74154047	c.350T>G	p.Leu117Trp	0	Uncertain Significance (PM1, PM2, PP3)	NA	Damaging	P1	novel
	-	chr14-74156171	c.485G>A	p.Trp162Ter	0	Uncertain Significance (PM2, PM4, PP3)	0.9946	Damaging	P1	novel
***LRRC6***	rs200906172	chr8-133673857	c.27T>G	p.Ile9Met	0	Uncertain Significance (PM2, BS4)	0.9907	Damaging	P2	ClinVar 473106
***DNAH11***	-	chr7- 21788220	c.8533C>G	p. Arg2845Gly	0.13	Likely benign (PM1, PM2, PP5, BP1, BP4, BP6)	0.9813	Damaging	P3	ClinVar 359658
***SPAG16***	rs61752199	chr2-214354811	c.1067G>A	p.Ser356Asn	2	Uncertain Significance (PM1, BS1)	0.9964	Damaging	P8	novel
	-	chr2-214174834	c. 331G>A	p.Asp111Asn	0	Uncertain Significance (PP3)	0.9993	Damaging	P11	novel
***SPAG17***	rs17185492	chr1-118644524	c. 473A>T	p.Glu158Val	17	Benign (PP2, BS1, BA1,)	0.9927	Damaging	P8, P11	novel

All identified genetic variants were numbered based on cDNA reference sequences and as recommended by the Human Genome Variation Society (http://www.hgvs.org/mutnomen). RefSeq accession numbers for the sequences used in the analyses were as follows: NM_017950.3 (*CCDC40*), NM_012144.2 (*DNAI1*), NM_001369.2 (*DNAH5*), NM_031427.3 (*DNAL1*), NM_001277115.1 (*DNAH11*), NM_012472.3 (*LRRC6*), NM_024532.4 (*SPAG16*), and NM_206996.2 (*SPAG17*).

^a^ According to VarSome (1000 Genome Project, ExaC)

^b^ ACMG: American College of Medical Genetics and Genomics.

^c^ Deleterious annotation of genetic variants using neural networks (DANN). The value range is from 0 to 1, with 1 given to the variants predicted to be the most damaging.

^d^ SIFT (sorts intolerant from tolerant) is an in silico prediction tool based on sequence homology derived from closely related sequences collected through PSI-BLAST. Provean (Protein Variation Effect Analyzer) is an in silico tool that predicts how nonsynonymous or in-frame indel variant will affect a protein's biological function.

^e^ This variant does not have automated ACMG/AMP 2015 interpretation

### RT-qPCR and Western blot analysis

**Fig 1:**
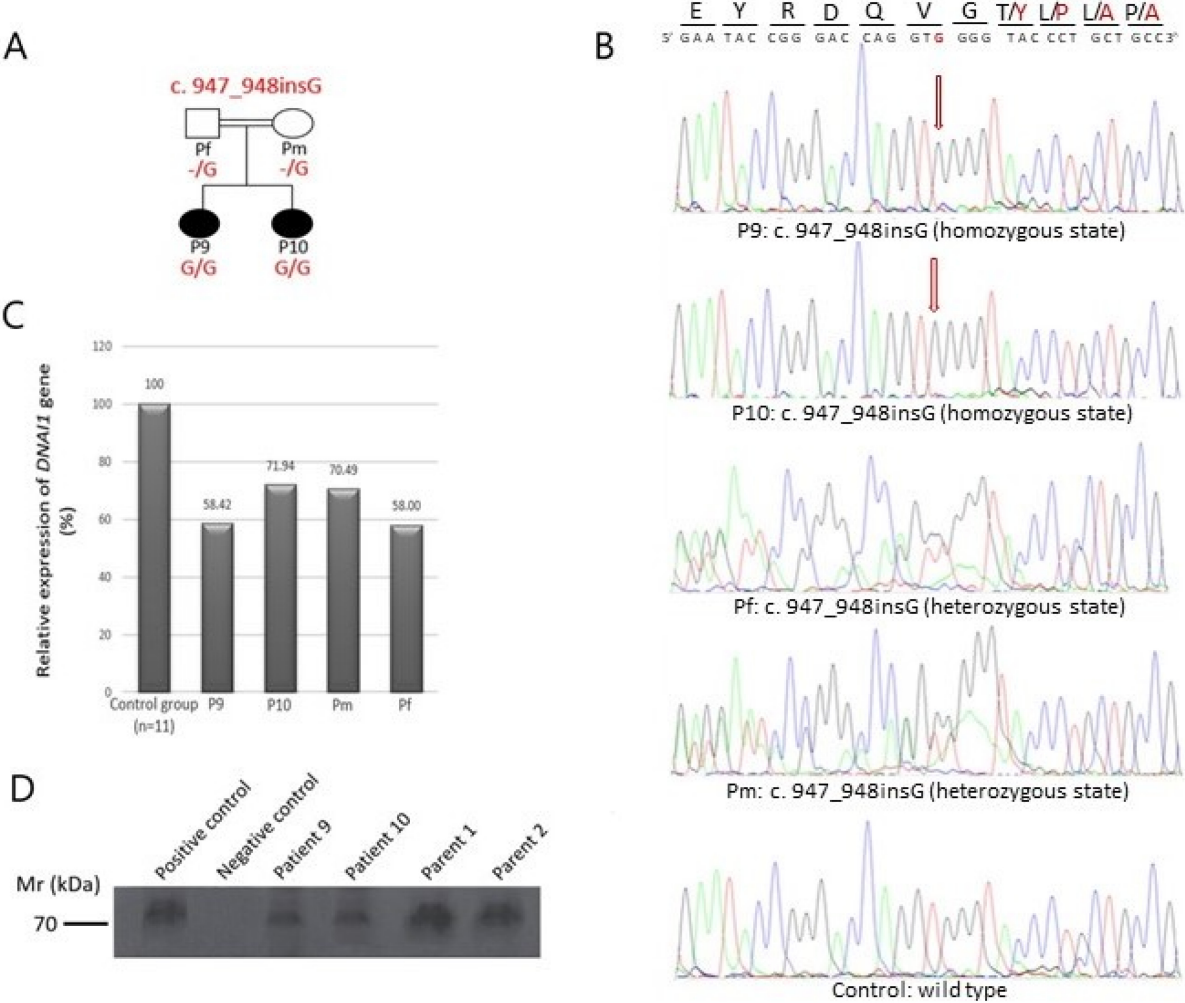
Confirmation by direct sequencing, expressional analysis and protein analysis of novel variant, c.947_948insG, found in *DNAI1* gene. (A) Pedigree structure of one PCD family with two affected sisters. (B) Sanger sequence confirmation of the 1 bp insertion (NM_012144.2: c.947_948insG) in exon 9 of *DNAI1* gene. First two sequences designated as P9 and P10 originated from the affected patients and displayed homozygous insertion of G nucleotide. Second two sequences (Pf and Pm) belongs to their parents and displayed insertion of G nucleotide on the one allele, and the last sequence originated from healthy control. For direct sequencing reverse primer was used, and therefore the sequences are aligned from the reverse side. (C) Comparison of the RT-qPCR profile of control group (mean of healthy control samples represents 100% expression), affected siblings (P9 and P10) and their parents (Pm and Pf). Relative expression of the DNAI1 transcript was 30–45% lower in two affected patients and their parents compared to healthy controls. (D) Immunoblotting of proteins from two patients with novel genetic variant, their parents and healthy control with rabbit Anti-Dynein intermediate chain 1 antibody, which reacts with human DNAI1 protein. Using Western Blot method we detected full length protein in patients (lanes 3 and 4), but the amount was lower in comparison to parents (lanes 5 and 6) and positive control (lane 1). Lane 2 represents negative control. The blot shown is representative of three independent experiments.

Among patients with detected variants in PCD causative genes, we found a novel homozygous variant in *DNAI1* gene (c. 947_948insG, p. Thr318TyrfsTer11), (GenBank: NM_012144.2), in two affected siblings (designated as P9 and P10). Their parents were heterozygous carriers of detected variant (Fig 1A and 1B).We analysed DNAI1 mRNA from peripheral blood mononuclear cells using RT-qPCR to investigate the potential effect of the nucleotide insertion on transcript stability. We found that the DNAI1 transcript was expressed 30–45% lower in two affected patients and their parents compared to controls (Fig 1C). DNAI1 protein was analysed using Western Blot method to examine the impact of frameshift stop mutation on the protein level, and full-length protein in patients was detected (Fig 1D, lanes 3 and 4). The amount of detected protein in patients was lower in comparison to parents (Fig 1D, lanes 5 and 6) and positive control (Fig 1D, lane 1).

### Computational analyses

**Fig 2:**
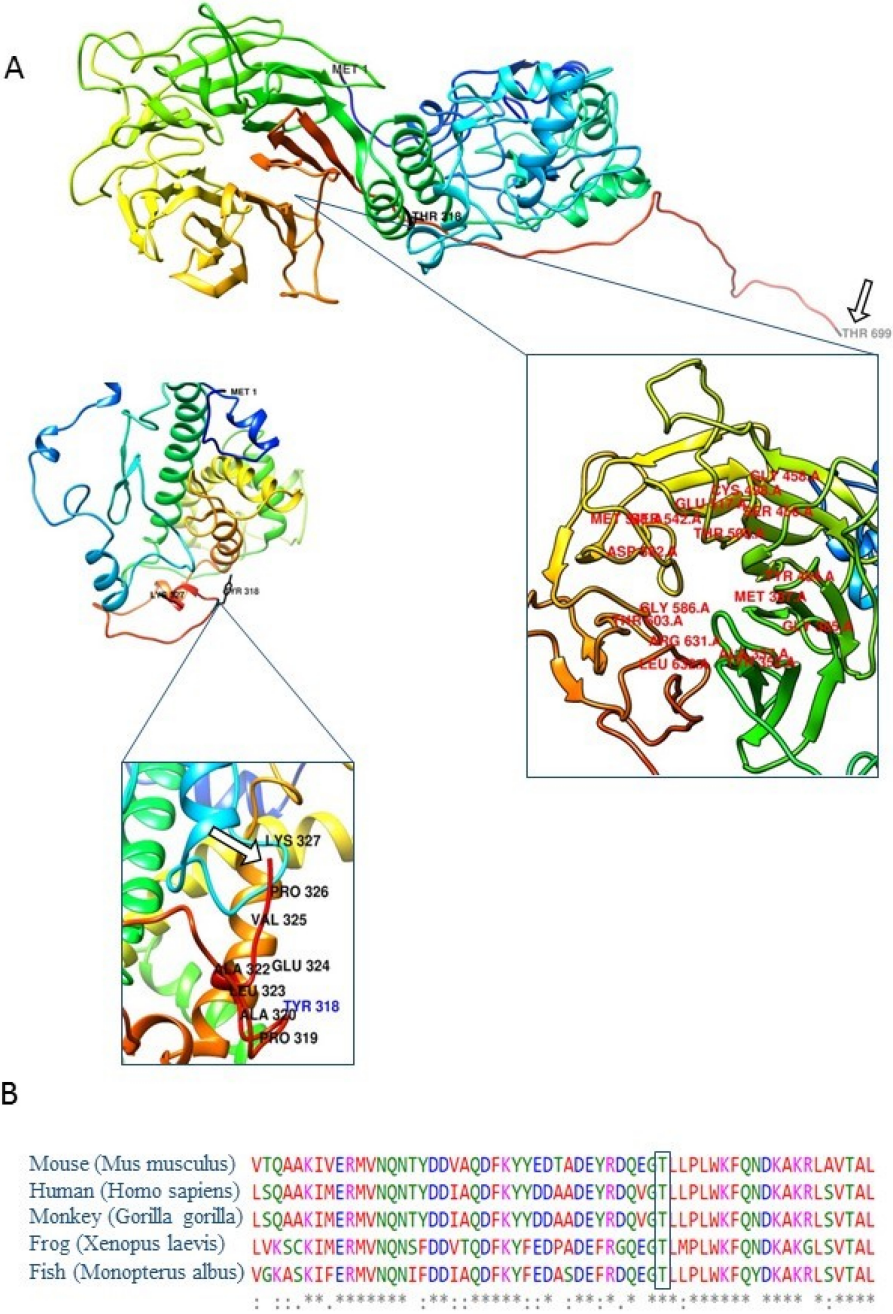
Three-dimensional molecular models of DNAI1 protein with close-up views of the regions harbouring novel frameshift genetic variant, and regions for PPIs. (A) View of the wild type (top picture) and mutated (bottom picture) DNAI1 protein. Wild type DNAI1 protein consists of 699 amino acids (GenBank accession number: NP_036276.1). Mutated is shorter than wild type and consists of 327 amino acids. Proteins begin to differ after position 318 in polypeptide chains. White arrows point to the last amino acid in polypeptide chains (Thr 699 in wild type and Lys 327 in mutated protein). Labelled amino acids in close-up view of wild type protein are representing ligand binding sites. Close-up view of mutated protein is displaying the altered region and truncation of protein due to frameshift stop mutation. Insertion has led to changes in open reading frame and consequently incorporation of incorrect amino acids downstream of insertion and premature stop codon. Images were prepared using UCFS Chimera software (http://www.rbvi.ucsf.edu/chimera). (B) Amino acid sequence alignment with ClustalW2. Five sequences were aligned and results indicate to high evolutionary conservation of residues affected by p.Thr318TyrfsTer11 variant among the DNAI1 orthologs in all analyzed vertebrate species (mouse, monkey, frog, fish and human). The amino acid marked with a rectangle displayed absolute evolutionary conservation among analysed species.

*In silico* modelling implied that amino acid change and consequential downstream introduction of multiple stop codons led to protein truncation (Fig 2A). We identified positions and amino acids in polypeptide chain participating in PPIs and established that the region in DNAI1 protein for PPIs is located downstream of premature stop codon (S3 Table). If for some reason, this mutated protein still exists in cells, its function is disrupted due to the absence of accurate amino acids in targeted position for PPIs (Fig 2A). Amino acid sequence alignment indicated high evolutionary conservation of residues affected by this variant among the DNAI1 orthologs in all analyzed vertebrate species (Fig 2B).

## Discussion

In the present study, we analysed the largest PCD cohort from Southeastern Europe ever reported. This is the first study which includes the genetic and molecular profiling, as well as phenotypic characterisation of PCD patients of Serbian ethnicity. Although large cohorts of PCD patients have been reported in Europe, only a small number of studies included molecular analysis of PCD patients [17, 20, 21, 27]. More often they included individual families or analysis of specific genes [22, 25, 28, 29]. Usage of custom-made population-specific platforms is convenient for detailed analysis of known PCD causative genes, but omits to detect novel candidate genes. Such limitations are circumvent by usage of whole-exome or whole-genome approaches. The approach presented in this study enables analysis of all exons of genes reported to be related to some human disorder, and thus enables discovery of potential candidate genes for PCD. The main limitation of this panel is the lack of all known PCD related genes, which can result in unresolved cases and lack of final genetic diagnosis.

We established the genetic cause of 9 (50%) unrelated patients involved in this study using NGS approach, which is consistent with the results published earlier [18, 21]. The most frequently mutated gene in our population, so far, is *DNAH5* (27.77%), which is consistent with results of previous studies on European population [21, 30]. Homozygous genetic variants were not detected in *CFTR* gene, as well as, presence of *Pseudomonas aeruginosa* and elevated chloride in sweat, which enabled us to exclude CF as potential diagnosis of analysed patients (Table 1).

*DNAI1*, *DNAH5*, *DNAH11*, and *DNAL1* genes are part of outer dynein arms (ODA)[31], and mutations in these genes were detected in 55.55% of our patients. Mutations in *LRCC6*, *SPAG16* and *SPAG17* genes found in 3 patients are responsible for combined defects in outer and inner dynein arms (ODA/IDA). Mutations in *CCDC40* gene lead to defects in IDA and nexin-dynein regulatory complexes (N-DRCs)[32]. To the best of our knowledge, this is the first report of genetic variants in *SPAG16* and *SPAG17* genes detected in PCD patients, although these genes are correlated with some symptoms in the literature [33]. *SPAG 17* encodes for central pair protein present in the "9 + 2" axonemes. The encoded protein is required for the proper function of the axoneme. Mutations in the orthologous gene in mice lead to PCD characterized by immotile nasal and tracheal cilia, reduced clearance of nasal mucus, respiratory distress, and neonatal lethality due to impaired airway mucociliary clearance [33]. The *SPAG16* gene encodes two major transcripts, SPAG16L and SPAG16S. This two proteins associate with the axoneme of sperm tail and the nucleus of postmeiotic germ cells, respectively [34]. Previous studies suggested that SPAG17, SPAG6, and SPAG16L form an interactome in the mammalian central apparatus. The significance of these interactions is inferred from the fact that deficiency in two of these proteins result in defects in spermatogenesis, sperm motility, and ciliary dysfunction [35].

### Characterization of homozygous genetic variant

Among patients with detected genetic variants in PCD causative genes, we found new potentially pathogenic variant (c.947_948insG located at exon 9 in *DNAI1* gene), presented in Axonemal dynein intermediate-chain gene (*DNAI1* (OMIM #604366)) was the first reported gene in which mutations were found to be associated with PCD, and so far 73 genetic variants were detected, according to ClinVar. Segregation analysis confirmed that homozygous variant in *DNAI1* gene that has been found in two siblings, was consistent with recessive inheritance of the mutation (Fig 1). This c.947_948insG genetic variant leads to a frameshift and an introduction of a UGA stop codon after 33 nucleotides, which would lead to synthesis of truncated protein of 327 amino acids (Fig 2). On the transcription level, we showed that DNAI1 mRNA transcript containing a premature stop codon is less expressed than wild type transcript (Fig 1C). This c.947_948insG transcript is most likely marked for degradation by nonsense-mediated mRNA decay, but the efficiency of this process is different in different cell types, and therefore some amount of this transcript remains in cells (Fig 1C, lanes: P9, P10, Pm, and Pf). Analysis of protein containing p.Thr318TyrfsTer11 amino acid change using Western Blot method surprisingly showed full-length protein in patients (Fig 1D, lanes P9, and P10) regardless of the presence of premature stop codon. In order to explain such result, we translated wild type and mutated mRNA sequence using Translate Tool (http://web.expasy.org/translate/) and discovered that this frameshift mutation leads to insertion of sixteen UGA stop codons downstream of premature UGA stop codon (S1 and S2 Figs). While the three stop codons typically lead to termination of translation, the process of protein synthesis termination, although effective, is not 100% efficient. Several natural mechanisms of termination suppression exist, including ribosomal frameshifting [36] suppressor tRNAs [37] and high-translational UGA readthrough, which we assume to be responsible for the presence of full length protein in our patients. Additionally, a selenocysteine tRNA can insert a selenocysteine (Sec) instead UGA stop codons, if a selenocysteine insertion sequence (SECIS element) is present in the untranslated regions [38, 39]. Although the occurrence of selenoprotein genes is limited, the Sec UGA codon has become the first addition to the universal genetic code.

We analysed the possibility of presence of the SECIS element in 3’UTR region of DNAI1 mRNA. Analysis of 60 nucleotides downstream of the originally stop codon for potential RNA secondary structures using mfold online program (http://unafold.rna.albany.edu/) [40] showed that dG of DNAI1 downstream sequence is in the range of the dG of downstream sequences of human selenoprotein P (SelP) gene (dG DNAI1: -14kcal/mol; dG SelP: -2.2kcal/mol to-16.6kcal/mol) [41]. This is an important finding since SelP contains multiple UGA codons [42], as well as DNAI1. Relying on previous studies on selenocysteine, our *in silico* findings, as well as high evolutionary conservation of this part of DNAI1 protein among ortholog species, we postulate a hypothesis that, for some reason, c.947_948insG mutation prefers the synthesis beyond the stop codon and insertion of selenocysteins for UGA stop codons rather than degradation of protein but biological significance of UGA recoding in DNAI1 protein still remains unclear.

### Genetic diagnosis of PCD, concluding remarks and future perspectives

There is currently no “gold standard” test to diagnose PCD [43]. PCD diagnostic procedures are complex, require expensive infrastructure, and an experienced team of clinicians, geneticists and microscopists [43–45]. Molecular diagnostics is a useful tool, especially in diseases with overlapping clinical presentation. Nevertheless, due to genetic heterogeneity of PCD, approximately 35% of PCD cases still lack confirmation of genetic basis of the disease [46]. NGS approach allows us to generate an extensive data libraries in short period of time and to analyse many genetic variants and potential candidate genes for PCD. Analysis of genetic background of PCD will lead to better understanding of the diseases and, eventually, to the design of new, molecular-targeted therapy.

## Supporting information

S1 Fig Translation of mutated mRNA sequence. Frameshift mutation led to premature stop codon (marked with red rectangle) and a consequentially insertion of sixteen stop codons. Codon for all seventeen stops was UGA (DNAI1 mRNA: NM_12144.3).
(PDF)

S2 Fig Nucleotide sequence alignment with ClustalW2. Two sequences were aligned from TGA stop codon and results indicate to evolutionary conservation of 53.3% among the mutated DNAI1 mRNA and SelP mRNA transcripts.
(PDF)

S1 Table Primers designed for validation of detected disease-causing mutations found in our cohort of PCD patients. (PDF)

S2 Table Primers designed for RT-qPCR analysis of *DNAI1* gene. (PDF)

S3 Table Positions of amino acids in DNAI1 protein essential for protein-protein interactions. (PDF)
