# Supplementary material for: Genomic profiling supports the diagnosis of primary ciliary dyskinesia and reveals novel candidate genes and genetic variants
Source: PLoS One. 2018 Oct 9;13(10):e0205422. doi: 10.1371/journal.pone.0205422 (PMC6177184; doi:10.1371/journal.pone.0205422)
Supplement: S1 Fig — Frameshift mutation led to premature stop codon (marked with red rectangle) and a consequentially insertion of sixteen stop codons. Codon for all seventeen stops was UGA (DNAI1 mRNA: NM_12144.3). (PDF) [file pone.0205422.s001.pdf]

Met I P A S A K A P H K Q P H K Q S I S I G R G T R K R D E D S G T E V G
E G T D E W A Q S K A T V R P P D Q L E L T D A E L K E E F T R I L T A N
N P H A P Q N I V R Y S F K E G T Y K P I G F V N Q L A V H Y T Q V G N L
I P K D S D E G R R Q H Y R D E L V A G S Q E S V K V I S E T G N L E E D
E E P K E L E T E P G S Q T D V P A A G A A E K V T E E E L **Met** T P K Q P
K E R K L T N Q F N F S E R A S Q T Y N N P V R D R E C Q T E P P P R T
N F S A T A N Q W E I Y D A Y V E E L E K Q E K T K E K E K A K T P V A K
K S G K **Met** A **Met** R K L T S **Met** E S Q T D D L I K L S Q A A K I **Met** E
R **Met** V N Q N T Y D D I A Q D F K Y Y D D A A D E Y R D Q V G Y P A A A
L E V P K **Stop** Q S Q A P V R H C P L L E S K V Q G S V C S G I W L L **Stop** L
H E A E P G H A A A L Q P E E P Q L P **Stop** V H V Q Q Q R R H V S R H
P R G P P L P G G S R P L **Stop** R Q R G H L Q P Q E A P L P A L L L Q L S
Q V W Q A L R P C V A G Q V A E G **Stop** H G P K P **Stop** L L L C V I **Stop** R
Q D C V L D S R E E K A G S H R C H Q A E G G R Q H H G S S **Stop** G V A
A A P S G L W H C L **Stop** L P Q R D **Stop** L H V P S G H R G G K N L Q V
L **Stop** I L L Q P I P R H L **Stop** R P Q H V S G H C V L E P I P H Q G L H V L
Q L R L D S E D L G P H H Q D P D V H L **Stop** P E L S R G **Stop** C G L G A
I L F Y C V R S S H H R W E G P H I **Stop** L S H Q Q V **Stop** G H L Q P A C
G G Q K E Q A H P R A V Q S H P P H H H C G R **Stop**
