# Supplementary material for: Genomic profiling supports the diagnosis of primary ciliary dyskinesia and reveals novel candidate genes and genetic variants
Source: PLoS One. 2018 Oct 9;13(10):e0205422. doi: 10.1371/journal.pone.0205422 (PMC6177184; doi:10.1371/journal.pone.0205422)
Supplement: S2 Fig — Two sequences were aligned from TGA stop codon and results indicate to evolutionary conservation of 53.3% among the mutated DNAI1 mRNA and SelP mRNA transcripts. (PDF) [file pone.0205422.s002.pdf]

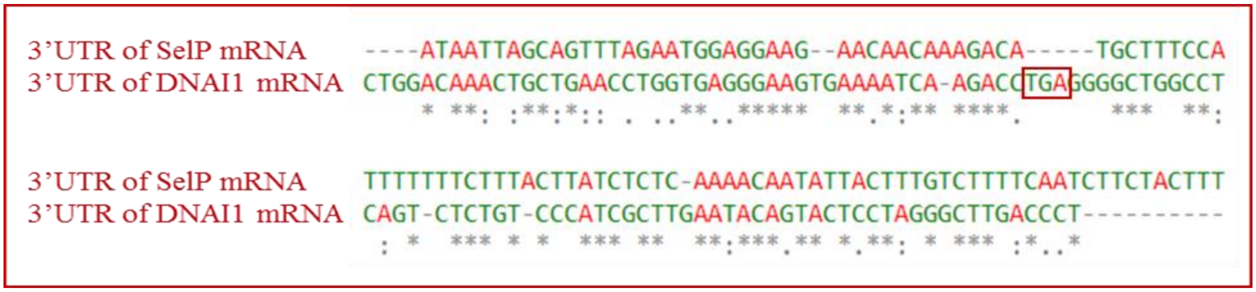
3’UTR of SelP mRNA
----ATAATTAGCAGTTTAGAATGGAGGAAG--AACAACAAAGACA-----TGCTTTCCA
3’UTR of DNAI1 mRNA
CTGGACAAACTGCTGAACCTGGTGAGGGAAGTGAAAATCA-AGACCTGAGGGGCTGGCCT
3’UTR of SelP mRNA
TTTTTTTCTTTACTTATCTCTC-AAAACAATATTACTTTGTCTTTTCAATCTTCTACTTT
3’UTR of DNAI1 mRNA
CAGT-CTCTGT-CCCATCGCTTGAATACAGTACTCCTAGGGCTTGACCCT----------
