# Supplementary material for: Genomic profiling supports the diagnosis of primary ciliary dyskinesia and reveals novel candidate genes and genetic variants
Source: PLoS One. 2018 Oct 9;13(10):e0205422. doi: 10.1371/journal.pone.0205422 (PMC6177184; doi:10.1371/journal.pone.0205422)
Supplement: S1 Table — (PDF) [file pone.0205422.s003.pdf]

Title of primers	Orientation	Sequence of primers	Length in bp
DNAL1_116/117F	Forward	5'-CTCCCACACAACTACTAGCTC-3'	21
DNAL1_116/117R	Reverse	5'-CTGTTTCTCTCTGAGCTTAGCC-3'	22
DNAL1_162F	Forward	5'-ATCTCCTGATGTCGTGATCCGC-3'	22
DNAL1_162R	Reverse	5'-CACCTTGGCTCAAACCTAGACC-3'	22
LRRC6_9F	Forward	5'-CAAAAACTCCAGGAGGAGGTACC-3'	23
LRRC6_9R	Reverse	5'-CACAGCACTTTCCAAAGTTCTCTC-3'	24
LRRC6_466F	Forward	5'-GGATGGGTGAGTTGAATCTG-3'	20
LRRC6_466R	Reverse	5'-GTTAAAACACTGGAGCAGCG-3'	20
DNAH5_379F	Forward	5'-GAGCTTCAAAGCAGCCATGTAG-3'	22
DNAH5_379R	Reverse	5'-CTCCCTCACATCCTTCCATTTC-3'	22
DNAH5_2542F	Forward	5'-GGGGAGAATTTGGAAGGTGAC-3'	21
DNAH5_2542R	Reverse	5'-GGATGCTTCATGTCTCCCC-3'	19
DNAH5_INTF	Forward	5'-GGAGGAGAGGAGCTTTTTGG-3'	20
DNAH5_INTR	Reverse	5'-GGGGTGAAAAGAGAACTTGGC-3'	21
CCDC40_83F	Forward	5'-CAGGTGTCACCACCAGAG-3'	18
CCDC40_83R	Reverse	5'-CTCTTGCTGGAAGCCTTGG-3'	19
SCNN1A_522F	Forward	5'-GCTGTTTCACCAAGTGCC-3'	18
SCNN1A_522R	Reverse	5'-CCTAGGAAAGAATGGGGGTG-3'	20
DNAH11_2600F	Forward	5'-CCACAGGGTGGATGAAATCG-3'	20
DNAH11_2600R	Reverse	5'-CCAACTTTCCAAAGATACAAAGGG-3'	24
DNAH11_2852F	Forward	5'-CCTGGGCAACAGAGCAAGTC-3'	20
DNAH11_2852R	Reverse	5'-GCCTTGGCCAAATCTGGGAG-3'	20
DNAI1_947_948F	Forward	5'-CCTGGGTTTGCCATAAAGCG-3'	20
DNAI1_947_948R	Reverse	5'-GTACTGGGGGTTTGGGATGTAGAG-3'	24
CCDC40_2440F	Forward	5'-CGGTCTAGGGGGAAGAAGTG-3'	20

CCDC40_2440R	Reverse	5'-GCAGGCAGTGGTTCACATTTAC-3'	22
DNAH5e13_F	Forward	5'-CAGTACCCGTGGAACCTGAC-3'	20
DNAH5e13_R	Reverse	5'-CTATGACCTTGTGACCCAAATTGCC-3'	25
DNAH5e54_F	Forward	5'-CATACGTATACGATAGAACAAAATCAGC-3'	28
DNAH5e54_R	Reverse	5'-CCCAATAGCACTTATTCACACCAC-3'	24
SPAG17e5_F	Forward	5'-CCTCATGCATAAATGTGCCTTGTCAC-3'	26
SPAG17e5_R	Reverse	5'-GCCACTGTGTTTCACTCGTGAAAG-3'	24
SPAG16e4_F	Forward	5'-GATAGGAGGACAGAGATTTGTTTGC-3'	26
SPAG16e4_R	Reverse	5'-GGTTTCAGGGCTGGAGATG-3'	19
CFTR22e_F	Forward	CCTGTTAGTTCATTGAAAAGCCCG	25
CFTR22e_R	Reverse	CTGCTTGCTTTGTTAGACTGTGTTC	24
SPAG16e10_F	Forward	5'-CAGAGTATCAACCTGTGCAGTG-3'	22
SPAG16e10_R	Reverse	5'-CTTAGTCTGTACTGCCACCAAG-3'	22
DNAH5e10_F	Forward	5'-CCCAAGCTCAAGTTAGAAGATTGTG-3'	25
DNAH5e10_R	Reverse	5'-GACATAGTAGGCTCCCAAGGAG-3'	22
DNAH5e49_F	Forward	5'-GGAAACACTCAAGGAGTTAGGCTC-3'	24
DNAH5e49_R	Reverse	5'-CAAGCAGGTGTTCTTCCTCACC-3'	22
DNAI1eg14_F	Forward	5'-GACAGAGATGGATGGAAGGAGGGAG-3'	25
DNAI1eg14_R	Reverse	5'-CTTTGCCATTTAGCTCCCTGCCTC-3'	24
DNAI1eg17_F	Forward	5'-CCAGGGAAGTGGTGGCTGC-3'	19
DNAI1eg17_R	Reverse	5'-CTGCAGAGGTGGAGGGTGGAAAG-3'	23
