# Supplementary material for: Genomic profiling supports the diagnosis of primary ciliary dyskinesia and reveals novel candidate genes and genetic variants
Source: PLoS One. 2018 Oct 9;13(10):e0205422. doi: 10.1371/journal.pone.0205422 (PMC6177184; doi:10.1371/journal.pone.0205422)
Supplement: S2 Table — (PDF) [file pone.0205422.s004.pdf]

Title of primers	Orientation	Sequence of primers	Length in bp
DNAI1_F_Exp	Forward	5'-GTCCCAAGCTGCTAAGATCATGGAGCG-3'	27
DNAI1_R_Exp	Reverse	5'-CAGGGTACCCACCTGGTCCC-3	20
