# Supplementary material for: Genomic profiling supports the diagnosis of primary ciliary dyskinesia and reveals novel candidate genes and genetic variants
Source: PLoS One. 2018 Oct 9;13(10):e0205422. doi: 10.1371/journal.pone.0205422 (PMC6177184; doi:10.1371/journal.pone.0205422)
Supplement: S3 Table — (PDF) [file pone.0205422.s005.pdf]

**Position**	337	352	385	387	404	436	456	458	498
**Residue**	A	Y	G	M	V	W	S	G	C
**Position**	500	517	541	542	562	586	603	631	633
**Residue**	T	E	M	S	D	G	T	R	T
